# Impact of reference body position on linear and 3D accuracy of intraoral scans

**DOI:** 10.1186/s40729-026-00686-2

**Published:** 2026-04-22

**Authors:** Alexander Schmidt, Haoyu Liu, Bernd Wöstmann, Maximiliane Amelie Schlenz

**Affiliations:** 1 Department of Prosthodontics, Christian Albrecht University of Kiel, University Hospital Schleswig-Holstein, Campus Kiel, Arnold-Heller-Straße 3, 24105 Kiel, Germany; 2 Department of Oral and Maxillofacial Surgery, University Hospital Giessen, Klinikstraße 33, 35392 Giessen, Germany; 3 Department of Prosthodontics, Dental Clinic, Justus Liebig University, Schlangenzahl 14, 35392 Giessen, Germany

**Keywords:** Intraoral scanners, Dental implants, Optical scanning, Three-dimensional imaging, Measurement accuracy, Reference standards, Digital dentistry, Dental prosthesis design, Dental models

## Abstract

**Purpose:**

Intraoral scanners (IOS) are widely used in digital implant workflows, and their accuracy is typically evaluated in vitro using reference bodies to establish a coordinate system. While the impact of scanning strategy and evaluation methods on accuracy is well documented, the potential effect of the spatial position of the reference body itself on measured transfer accuracy has not been systematically investigated yet. This study aimed to evaluate whether the position of a reference body within a master model affects linear and three-dimensional (3D) accuracy outcomes of intraoral scan data.

**Methods:**

A partially edentulous maxillary implant master model with four implants (FDI #16, #14, #25, and #26) and two perpendicular cuboid-shaped reference bodies was scanned ten times each using Trios 4 and Primescan AC IOS. Each dataset was evaluated twice by aligning it either to reference body 1 (REF-1) or reference body 2 (REF-2), generating paired measurements from identical scans. Linear distances between implant-abutment interface points and 3D deviations from the reference bodies were calculated. Trueness and precision were assessed according to ISO 5725-1, and paired statistical comparisons were performed. Differences between REF-1 and REF-2 were evaluated by paired t-tests for trueness and variance-related tests for precision, with a significance level set at α = 0.05.

**Results:**

For both scanners, significant differences in trueness were observed between REF-1 and REF-2 for several linear distances and three-dimensional deviations (*p* < 0.05), despite identical scan datasets. Reference-dependent effects were more pronounced for three-dimensional deviations than for linear measurements. For selected distances, significant differences in precision were also detected (*p* < 0.05).

**Conclusions:**

The position of the reference body within a model significantly influences measured linear and 3D implant accuracy, independent of the IOS system used. Alignment reference position represents an independent source of systematic bias in digital accuracy assessments. These findings should be considered when designing, interpreting, and comparing accuracy studies in digital implant dentistry.

**Graphical abstract:**

**Figure:**
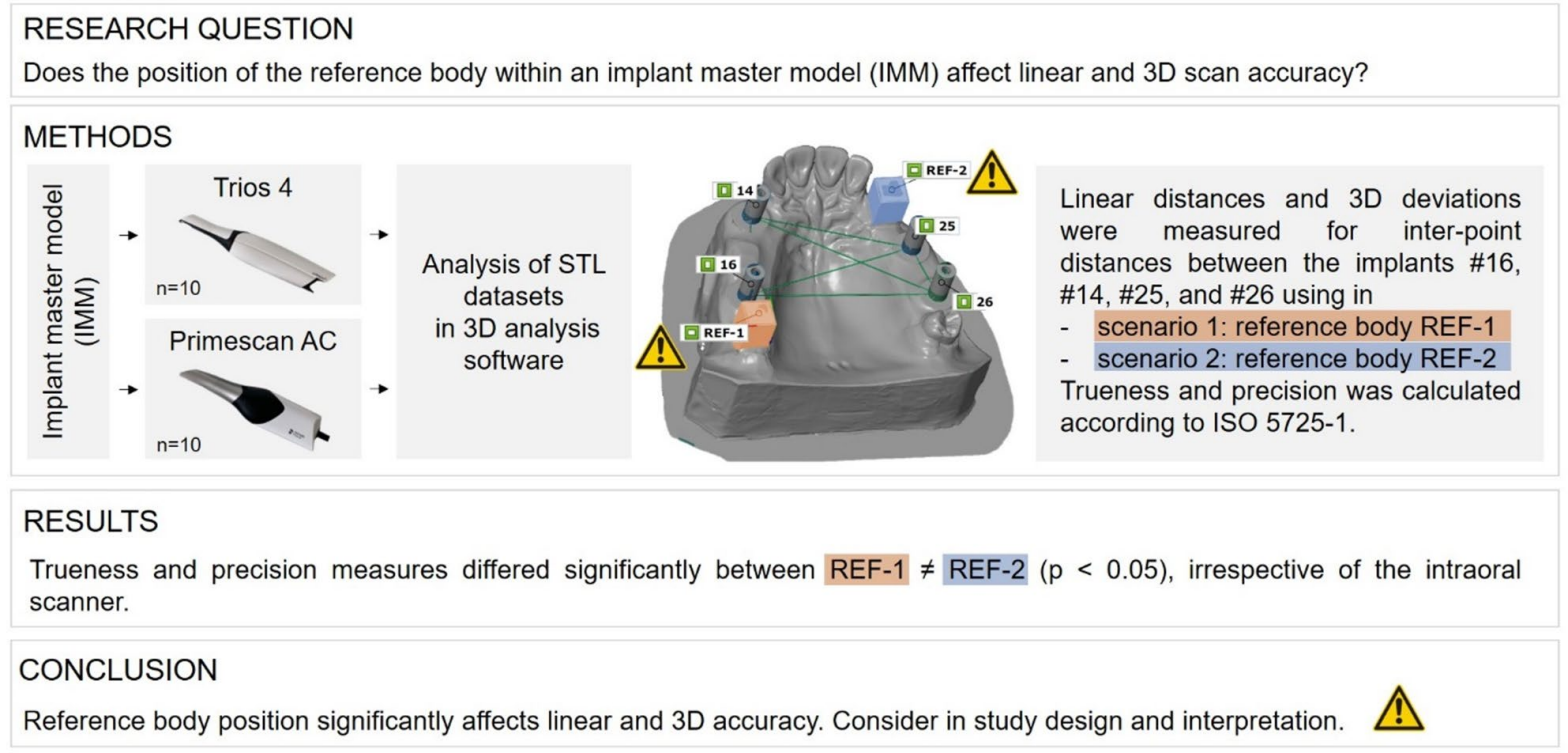
Influence of reference body position on linear and three-dimensional accuracy of intraoral scans

## Background

 The use of intraoral scanners (IOS) has increased substantially in recent years and has become a central component of modern digital workflows in implant dentistry, with numerous studies focusing on their accuracy. Typically, the transfer accuracy from the intraoral situation to the extraoral model is of primary interest. However, a completely error-free transfer is still not achievable to date [1, 2]. In this context, in vitro studies are more frequently conducted than in vivo studies [3], mainly because in vitro setups allow the integration of reference bodies, which are difficult to implement under clinical conditions. To date, only a few clinically applicable studies using reference structures have been reported [4–8].

The use of reference bodies is essential because, without them, potential transfer errors of unknown magnitude may occur when transferring the intraoral situation to an extraoral model. As a result, when comparing different impression techniques–such as conventional versus digital impressions–no valid conclusion can be drawn regarding which technique yields more accurate values if no reference structure is available [4, 7]. In contrast to in vivo settings, reference bodies can be implemented much more easily in in vitro experiments. Various configurations are possible regarding their geometry and position. Previous investigations have primarily employed cuboid-shaped reference bodies, which may be positioned in the palate or within the dentition [2, 9].

It is well established that the accuracy of IOS decreases with increasing scan path length due to cumulative matching or stitching errors [8, 10, 11]. However, the magnitude of these errors can vary substantially depending on the measurement and evaluation method used. Several studies have demonstrated that three-dimensional (3D) deviation analysis provides more metrically precise data compared with best-fit surface overlay methods, which may mask or redistribute local deviations [12–17]. This aspect is particularly relevant in implant dentistry, as implants exhibit significantly lower intrinsic mobility than natural teeth, described in the literature by a factor of ten [12, 18–20].

Despite this knowledge, it remains completely unclear how the positioning of a reference body within a model may influence measured transfer accuracy. This question is of major importance when comparing the results of different studies, especially if each study uses reference structures in different locations or geometries. To the best of the authors’ knowledge, no study has systematically investigated how the position of a reference body within the model itself affects the measured accuracy of intraoral scans.

Therefore, the aim of the present study was to integrate two cuboid-shaped reference bodies within a single master model and to evaluate the impact of reference body position on transfer accuracy within a digital implant workflow.

The following null hypothesis was defined: The positioning of a reference body within a model has no influence on transfer accuracy.

## Methods

### Implant master model

A previously fabricated partially edentulous maxillary model was used as the implant master model (IMM) to simulate a clinical scenario with an interrupted and a unilaterally shortened dental arch. The model was mounted on a 100 × 100 mm stainless-steel base plate containing four prefabricated metal cylinders representing the implant sites at FDI positions #16, #14, #25, and #26. Straumann RN Standard Plus implants (Straumann, Freiburg, Germany) with a length of 14 mm and a diameter of 4.8 mm were adhesively fixed into the cylinders using a resin-based luting material (Galvano AGC-Cem, Wieland-Dental, Würzburg, Germany).

**Fig. 1:**
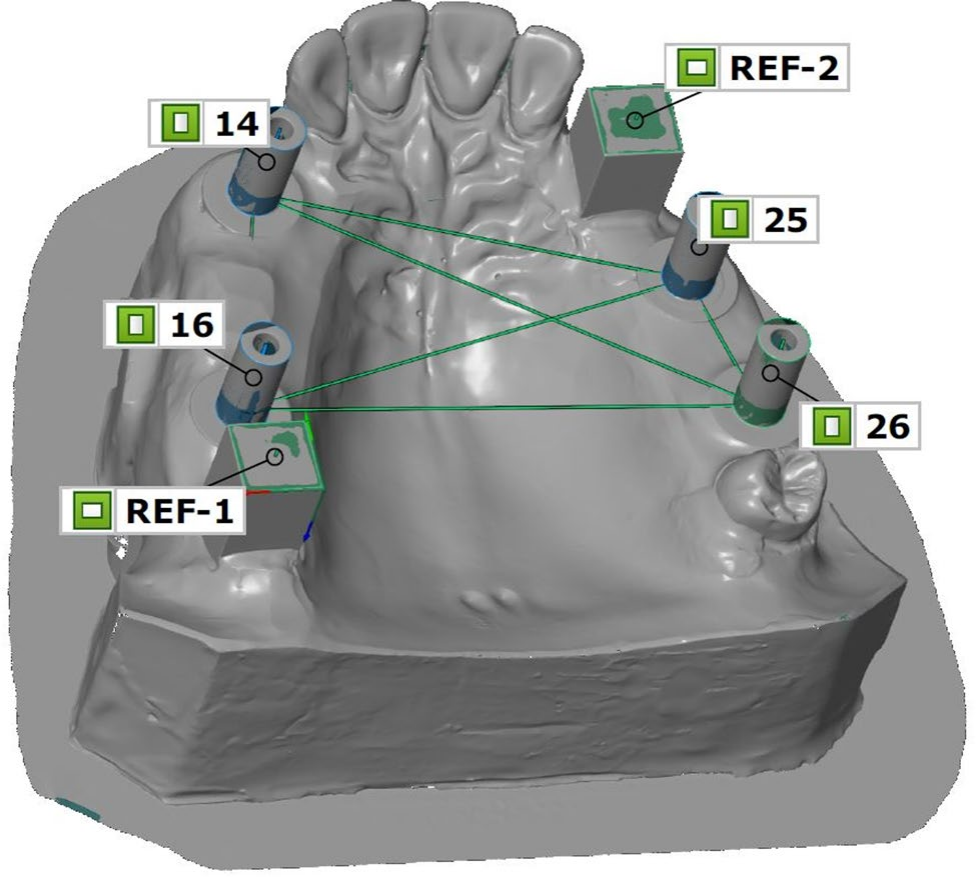
Constructed linear distances between the implant-abutment interface points (IAIPs) of the individual implants at FDI positions #16, #14, #25, and #26 in the implant master model (IMM), illustrated by the green connecting lines

Two geometrically defined cuboid-shaped reference bodies (REF-1 and REF-2) were integrated at sites corresponding to FDI positions #18 and #23. These cuboids were arranged perpendicular to one another, enabling the establishment of a reproducible 3D coordinate system for subsequent measurements. The anatomical contours of the partially edentulous maxilla were reproduced using a pink-colored methyl methacrylate resin (PalaXpress, Kulzer, Hanau, Germany). Figure 1 shows the final configuration of the IMM with all reference elements.

### Intraoral scanners and scanning procedure

Two widely used IOS were used for digital acquisition: Trios 4 (3Shape, Copenhagen, Denmark; software version 19.2.4) and Primescan AC (Dentsply Sirona, Bensheim, Germany; software version 5.1.0).

For each IOS, ten complete scans (*n* = 10) of the IMM were performed, resulting in a total of 20 digital datasets. All scans were acquired by a single experienced operator to avoid inter-operator variability (H.L.). The scan path followed the manufacturer-recommended scanning strategy for full-arch maxillary impressions, beginning in the posterior region and progressing anteriorly with continuous capture of palatal and buccal surfaces. Each resulting scan was exported in standard tessellation language (STL) format for further processing.

### Reference body assignment and dataset generation

To evaluate the potential influence of reference body position on measurement outcomes, each scan was analyzed twice: REF-1 assignment with registration and measurement using reference body 1 and REF-2 assignment with registration and measurement using reference body 2. This procedure generated paired datasets for each scan, allowing intra-scan comparison of the two reference body positions while keeping all other variables constant.

Two categories of deviations were assessed:

*Linear distances*

Distances were calculated between the defined reference points of the reference bodies and the implant-abutment interface points (IAIPs). The following inter-point distances were measured consistently across all datasets: FDI #16 to #14, #16 to #25, #16 to #26, #14 to #25, #14 to #26, and #25 to #26 (Fig. 1).

*3D deviations*

**Fig. 2:**
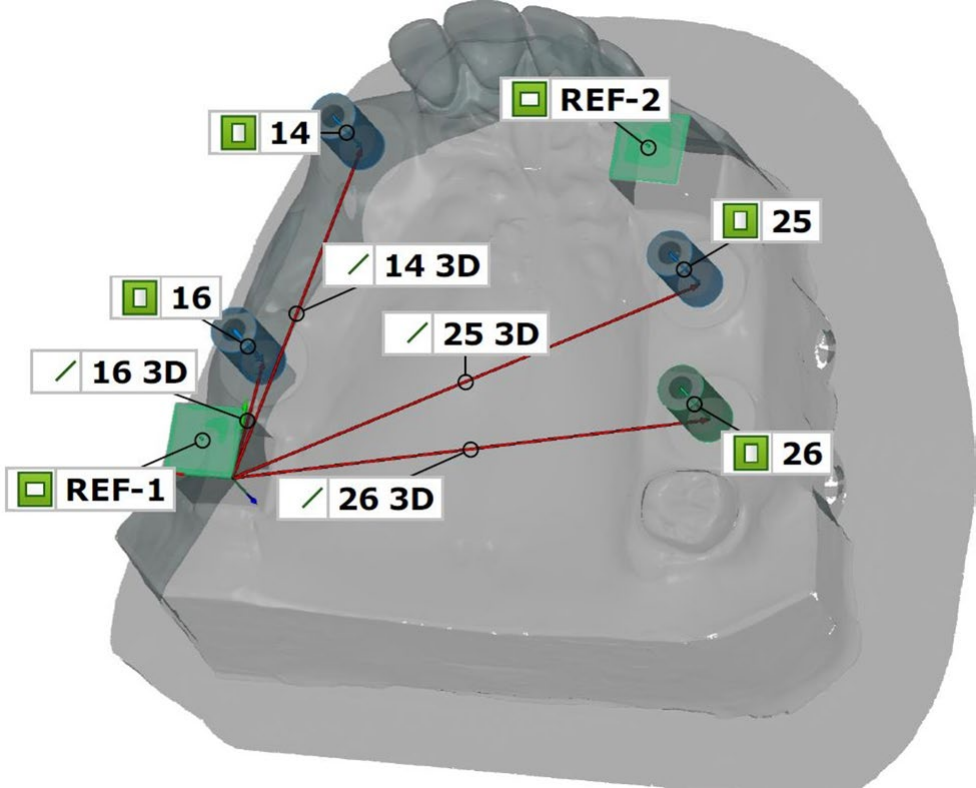
Three-dimensional deviations (ΔR) between the origin of the reference cuboid and the implant-abutment interface points (IAIPs) in the implant master model (IMM), illustrated by the red connecting lines. For reasons of clarity, the three-dimensional distances are schematically shown only for REF-1; the corresponding distances for REF-2 were calculated analogously but are not displayed

For each dataset, the three-dimensional (3D) deviation of the implant–abutment interface points (IAIPs) relative to the coordinate system defined by the respective reference body was calculated as an absolute Euclidean distance. The 3D deviations (ΔR) between the reference data set and the corresponding impression data were determined according to the following equation: ΔR= $\:\sqrt[2]{{\left( {x_{2} - x_{1} } \right)^{2} + \left( {y_{2} - y_{1} } \right)^{2} + \left( {z_{2} - z_{1} } \right)^{2} }}$ (Fig. 2).

### Data processing and alignment

All STL datasets were imported into a 3D analysis software (GOM Inspect, Zeiss, Braunschweig, Germany; version 2019).

Each scan was aligned to the IMM reference coordinate system using either: REF-1 or REF-2, depending on the evaluation condition. The alignment was carried out through local registration to the surfaces of the cuboid-shaped reference body, after which linear distances and 3D deviations were computed. The alignment procedure was performed using a standardized workflow within the software and did not require user-defined seed point selection. The same registration protocol was applied consistently to all datasets.

This approach enabled a direct comparison of measurement outcomes based solely on the position of the reference body used for alignment, without differences in scanning conditions.

### Statistical analysis

Data analysis was performed using SPSS Statistics (IBM, Armonk, NY, USA; version 31.0). For each scanner and each measured distance, datasets obtained using reference body 1 (REF-1) and reference body 2 (REF-2) were analyzed in a paired design. Trueness and precision were assessed according to ISO 5725-1 [21], with the mean deviation representing trueness and the standard deviation representing precision. Differences in trueness between REF-1 and REF-2 were evaluated using paired t-tests, while differences in precision were assessed using variance-related statistical tests for paired observations. The level of significance was set at α = 0.05.

## Results

### Linear distances between IAIPs

#### Trios 4

**Fig. 3:**
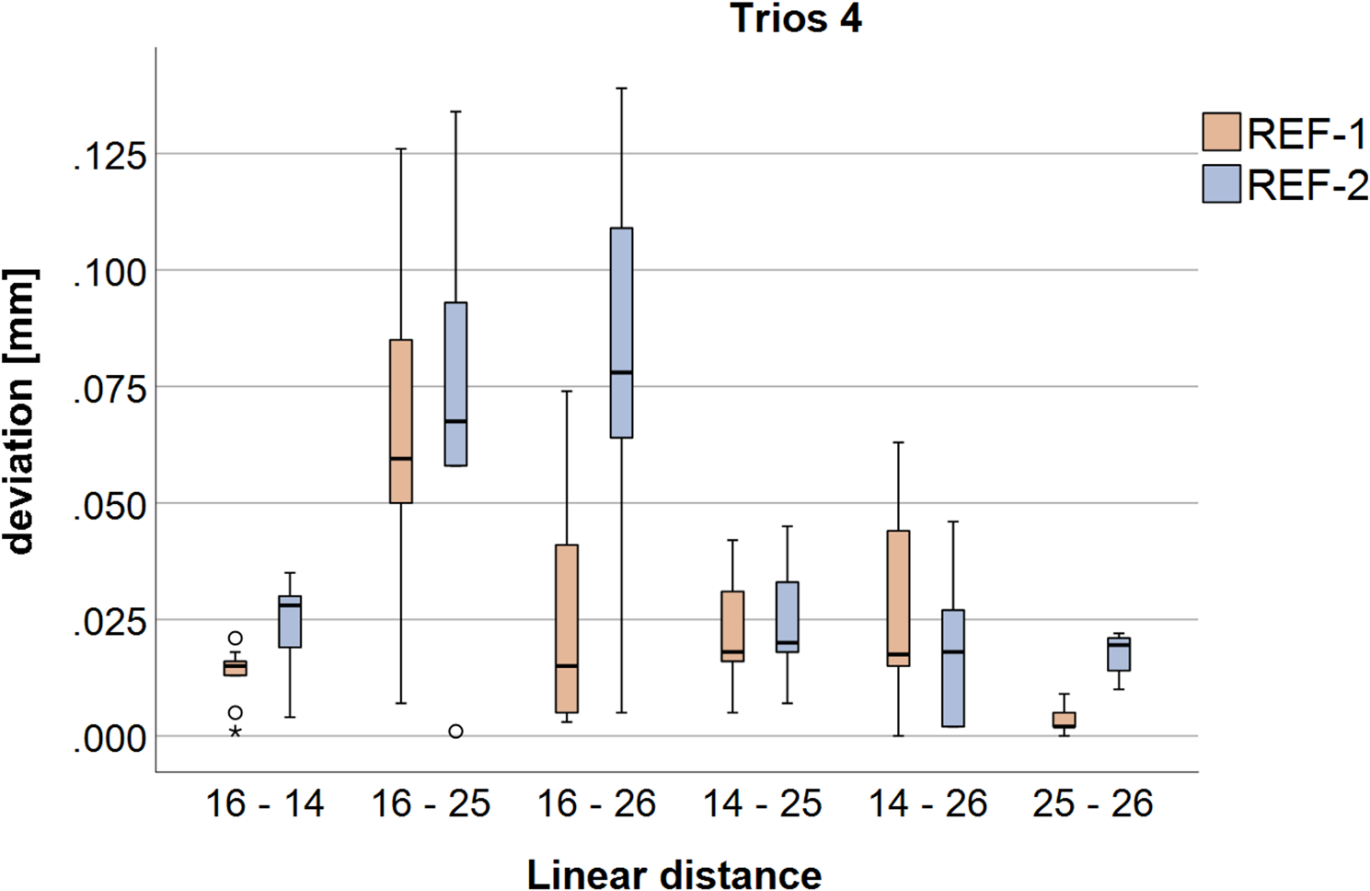
Boxplots of the linear distances between the implant-abutment interface points (IAIPs) at the individual implant positions (FDI #16, #14, #25, and #26) for Trios 4, shown separately for reference body 1 (REF-1) and reference body 2 (REF-2)

The results of the linear distances between the IAIPs obtained with the Trios 4 scanner are presented in Fig. 3; Table 1 according to ISO 5725-1 [21]. Significant differences (*p* < 0.05) between REF-1 and REF-2 are denoted by an asterisk.

**Table 1:** Linear IAIPs distances for Trios 4 and Primescan AC expressed as mean ± standard deviation (trueness and precision according to ISO 5725-1 [21]) for REF-1 and REF-2, including *p*-values for paired comparisons of trueness and precision

Intraoral scanner	Linear distance	Trueness/precision (mean [mm] ± standard deviation [mm])			
		REF-1 mean [mm] ± standard deviation [mm]	REF-2 mean [mm] ± standard deviation [mm]	*p*-value (trueness)	*p*-value (precision)
Trios 4	16–14	0.013 ± 0.006	0.025 ± 0.009	**0.005**	0.185
	16–25	0.064 ± 0.031	0.071 ± 0.034	0.656	0.914
	16–26	0.024 ± 0.024	0.083 ± 0.039	**0.001**	0.152
	14–25	0.021 ± 0.012	0.024 ± 0.012	0.697	0.965
	14–26	0.026 ± 0.021	0.017 ± 0.015	0.289	0.145
	25–26	0.003 ± 0.003	0.018 ± 0.004	**< 0.001**	0.106
Primescan AC		0.036 ± 0.007	0.051 ± 0.007	**< 0.001**	0.964
	16–25	0.020 ± 0.015	0.020 ± 0.016	0.955	0.870
	16–26	0.024 ± 0.024	0.024 ± 0.025	0.993	0.960
	14–25	0.026 ± 0.013	0.024 ± 0.012	0.752	0.973
	14–26	0.031 ± 0.017	0.048 ± 0.017	**0.033**	> 0.999
	25–26	0.009 ± 0.003	0.028 ± 0.003	**< 0.001**	> 0.999

Statistically significant differences (*p* < 0.05) are highlighted in bold type

Although identical scan datasets were analyzed, several distances showed significant differences in trueness depending on the reference body used for alignment, indicating an influence of the reference body position on the measured linear deviations. With regard to precision, variance-related testing revealed significant differences for selected distances, whereas other distances showed comparable repeatability.

#### Primescan AC

**Fig. 4:**
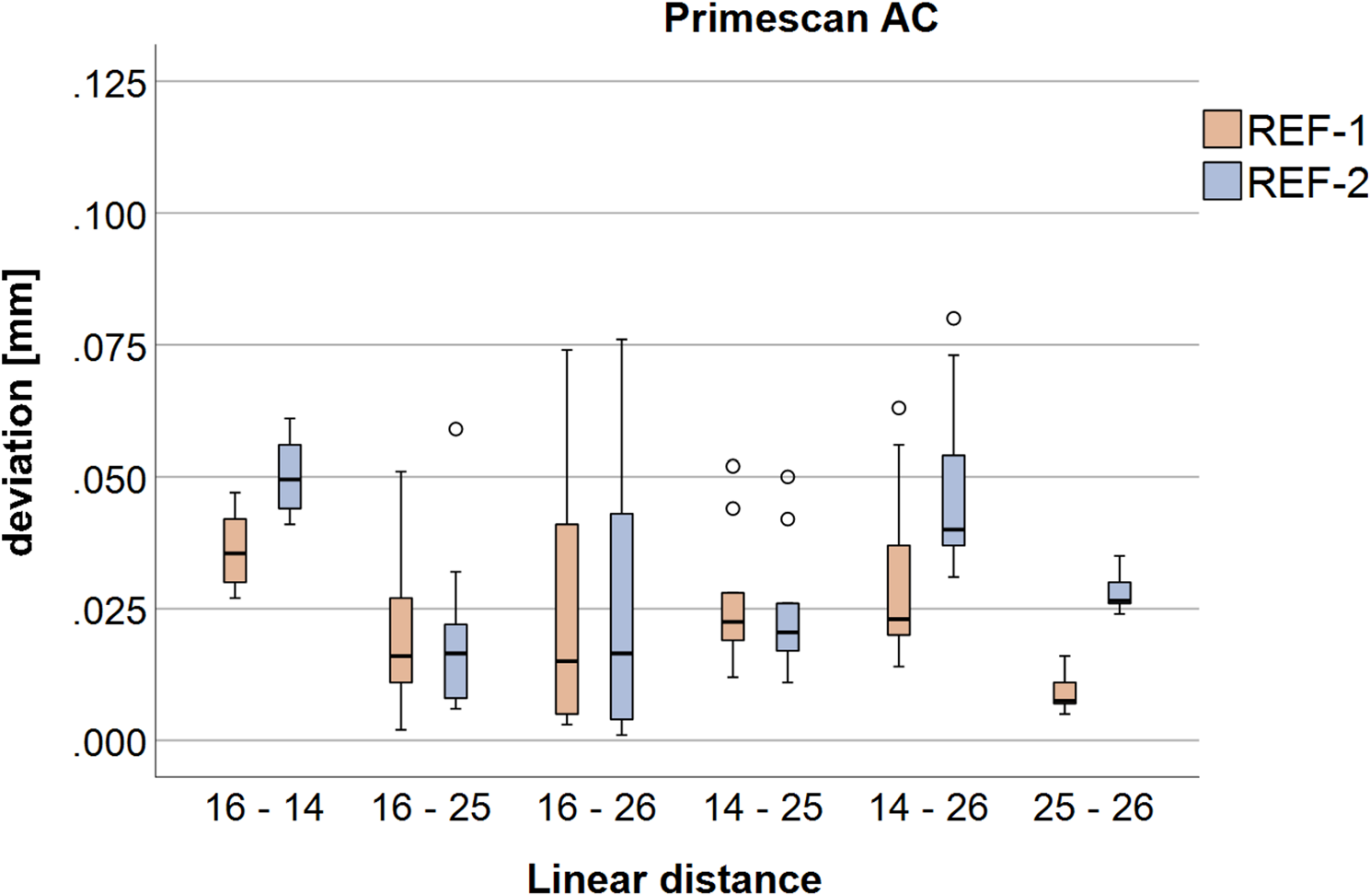
Boxplots of the linear distances between the implant-abutment interface points (IAIPs) at the individual implant positions (FDI #16, #14, #25, and #26) for Primescan AC, shown separately for reference body 1 (REF-1) and reference body 2 (REF-2)

The corresponding results for the IOS Primescan AC are shown in Fig. 4; Table 1.

Similar to Trios 4, significant differences in trueness between REF-1 and REF-2 were observed for several linear distances, despite identical scan datasets and identical IAIPs. These findings demonstrate that the measured linear deviations depend on the position of the reference body used for alignment. For precision, significant differences were detected for selected distances, while for the remaining distances no significant differences in repeatability were found.

### 3D deviations between IAIPs

#### Trios 4

**Fig. 5:**
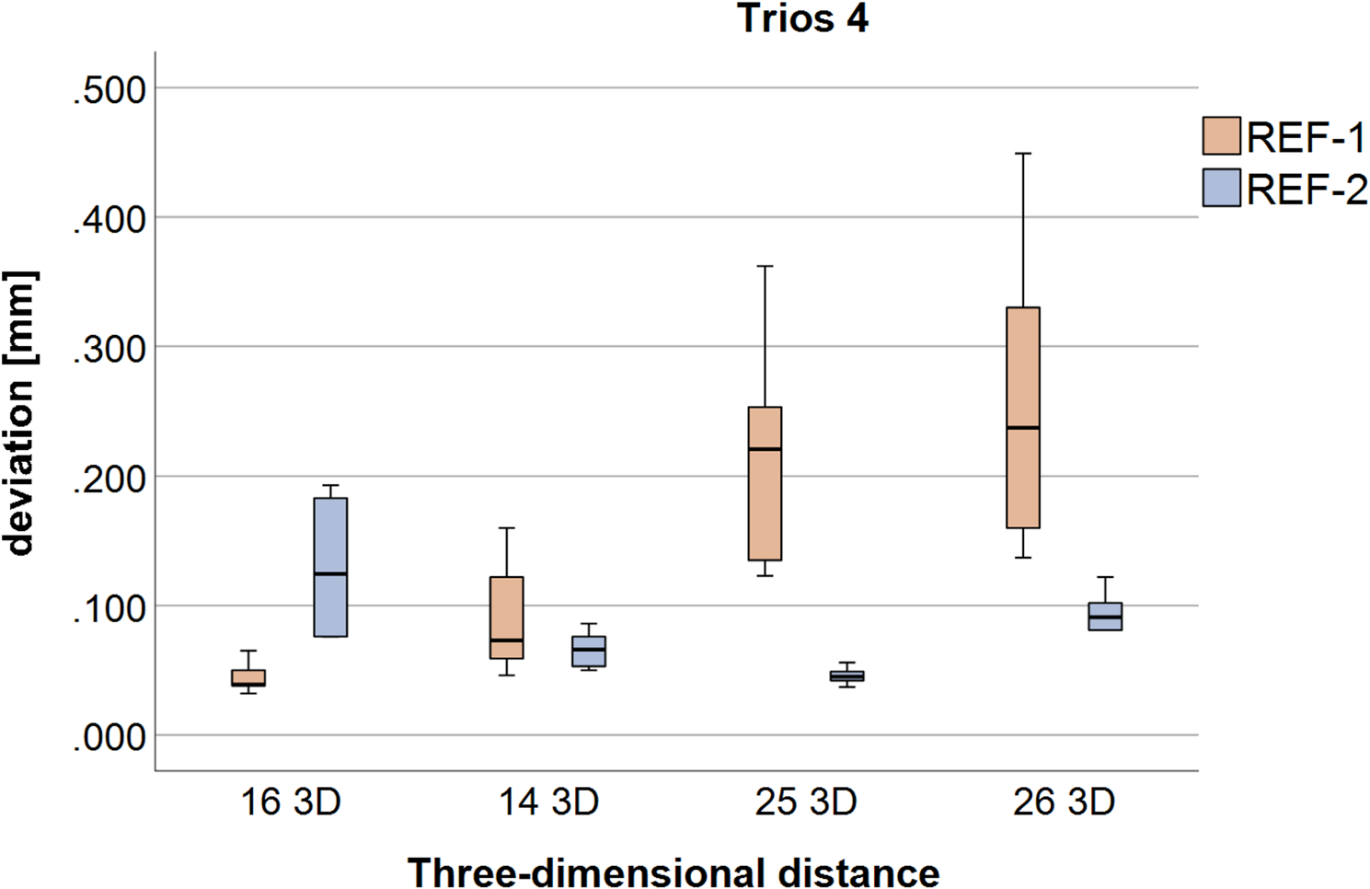
Boxplots of the three-dimensional deviations (three-dimensional (3D) deviations) between the reference body origin and the implant-abutment interface points (IAIPs) at the individual implant positions (FDI #16, #14, #25, and #26) for Trios 4, shown separately for reference body 1 (REF-1) and reference body 2 (REF-2)

The 3D deviations between the IAIPs obtained with the Trios 4 scanner are presented in Fig. 5; Table 2 according to ISO 5725-1 [21]. Significant differences (*p* < 0.05) between REF-1 and REF-2 are denoted by an asterisk.

**Table 2:** Three-dimensional (3D) implant-abutment interface points (IAIPs) deviations for Trios 4 and Primescan AC expressed as mean ± standard deviation (trueness and precision according to ISO 5725-1 [21]) for REF-1 and REF-2, including *p*-values for paired comparisons of trueness and precision

Intraoral scanners	Three-dimensional deviation (3D)	Trueness/precision (mean [mm] ± standard deviation [mm])			
		REF-1 mean [mm] ± standard deviation [mm]	REF-2 mean [mm] ± standard deviation [mm]	*p*-value (trueness)	*p*-value (precision)
Trios 4	16 3D	0.044 ± 0.011	0.132 ± 0.047	**< 0.001**	**< 0.001**
	14 3D	0.092 ± 0.043	0.066 ± 0.012	0.243	**0.001**
	25 3D	0.214 ± 0.072	0.045 ± 0.006	**< 0.001**	**0.003**
	26 3D	0.258±0.100	0.093 ± 0.014	**< 0.001**	**< 0.001**
Primescan AC	16 3D	0.038 ± 0.014	0.101 ± 0.048	**< 0.001**	**0.007**
	14 3D	0.085 ± 0.024	0.048 ± 0.030	**0.019**	0.735
	25 3D	0.115 ± 0.053	0.042 ± 0.010	**< 0.001**	**0.007**
	26 3D	0.110 ± 0.049	0.070 ± 0.005	**0.011**	**0.005**

Statistically significant differences (*p* < 0.05) are highlighted in bold

For several 3D distances, significant differences in trueness were observed between the two reference body positions, indicating that the reference body location affects not only linear but also 3D accuracy. With regard to precision, variance-related testing revealed significant differences for some distances, whereas others showed comparable repeatability.

#### Primescan AC

**Fig. 6:**
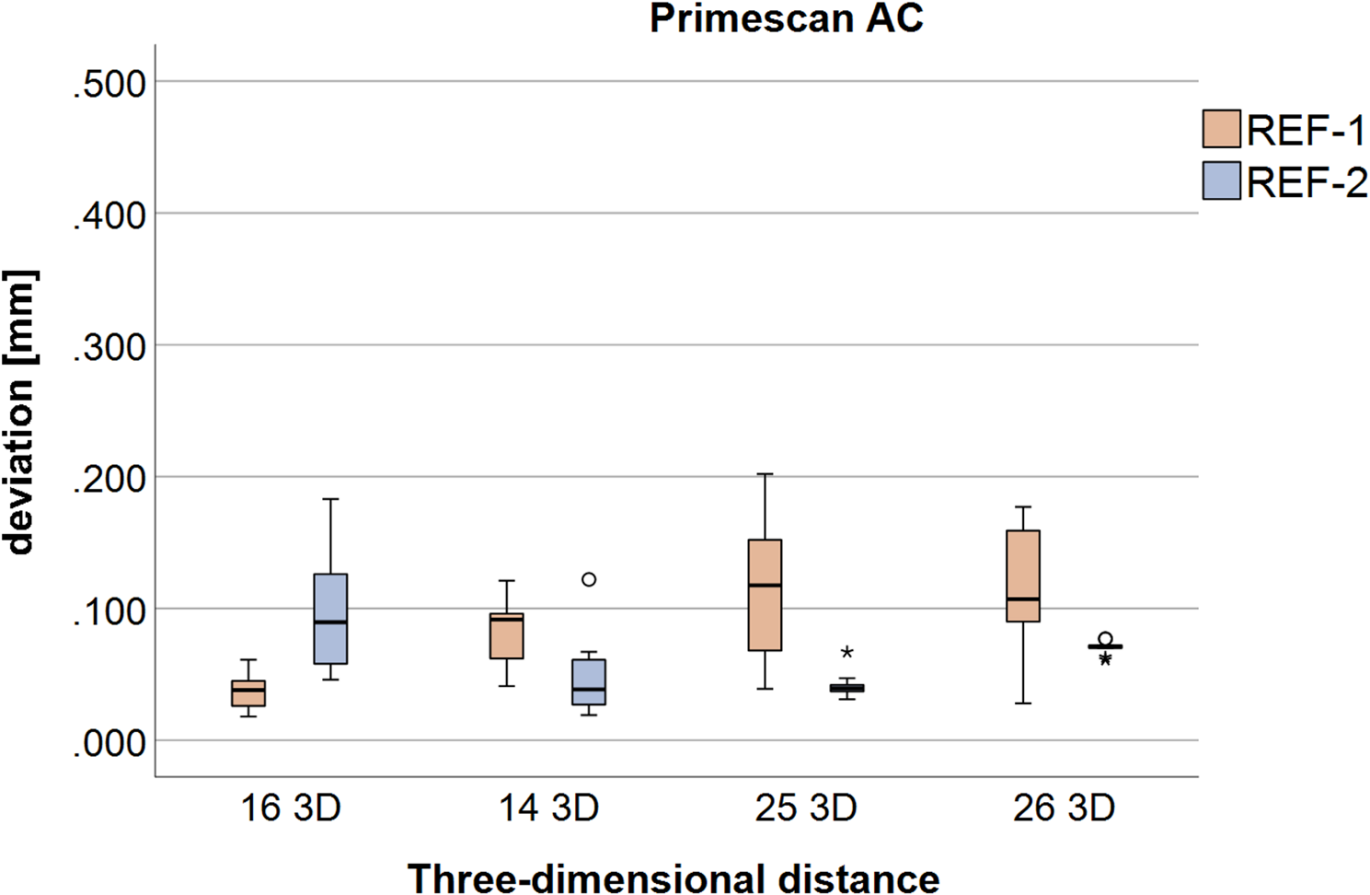
Boxplots of the three-dimensional deviations (three-dimensional (3D) deviations) between the reference body origin and the implant-abutment interface points (IAIPs) at the individual implant positions (FDI #16, #14, #25, and #26) for Primescan AC, shown separately for reference body 1 (REF-1) and reference body 2 (REF-2)

The 3D results for the Primescan scanner are shown in Fig. 6; Table 2.

Comparable to the Trios 4, significant differences in trueness between REF-1 and REF-2 were detected for several 3D distances, despite identical scan datasets being evaluated. This indicates that the 3D accuracy of the measurements is influenced by the position of the reference body used for alignment. For precision, significant differences were found for selected distances, while other distances showed no significant differences.

Overall, both linear and 3D measurements between the IAIPs demonstrated reference body-dependent differences in trueness and, in part, in precision for both IOS, the Trios 4 and the Primescan AC. Thus, even when identical scan datasets and identical measurement points are used, the position of the reference body within the model significantly influences the measured accuracy. Consequently, the null hypothesis that the positioning of the reference body within the model has no influence on transfer accuracy must be rejected.

## Discussion

The present in vitro study investigated whether the position of a reference body within a model influences the measured accuracy of intraoral scan data when identical datasets are analyzed. The main finding of this study is that both linear distances between IAIPs and 3D deviations are significantly affected by the selected reference body position. This effect was observed for both investigated IOS (Trios 4 and Primescan AC) and was evident for trueness and, for selected distances, also for precision. Thus, the alignment reference itself represents a relevant systematic factor in accuracy assessments, independent of the scanner system used. Although the absolute trueness and precision values obtained in the present study are in the range of those reported for Trios 4 and Primescan AC in previous in vitro and in vivo investigations [2, 8, 9, 22], the aim of the present investigation was not to compare the scanners themselves. Instead, both systems were included to demonstrate that reference body-dependent effects occur irrespective of the IOS system. Consequently, no direct inter-scanner comparison was intended.

### Linear versus 3D deviations

In the present study, 3D deviations revealed more pronounced and more consistent differences between REF-1 and REF-2 than linear distances. This finding is in accordance with previous observations that absolute 3D measurements are more sensitive to systematic spatial distortions than individual linear distances or best-fit overlays, which may partially mask accumulated registration errors [17, 23, 24]. Linear measurements describe deviations along single axes or between selected point pairs, whereas 3D Euclidean distances reflect the full spatial displacement of an object and therefore capture the cumulative effect of matching and stitching errors more comprehensively. Consequently, the observed larger differences in 3D deviations support the assumption that reference-dependent alignment effects propagate in all spatial dimensions and cannot be fully represented by isolated linear measurements alone.

### Influence of reference body position on matching and stitching

IOS generate 3D models by incremental acquisition and registration of overlapping point clouds. The alignment process is locally optimized based on geometric features within the currently acquired area, while small registration errors accumulate along the scan path and may lead to global distortions of the reconstructed model [22, 25, 26].

The reference body defines the coordinate system and acts as an anchor for the superimposition of the datasets. A reference body located closer to the region of interest minimizes the propagation distance of registration errors, whereas a reference body positioned further away increases the effective scan path length between the anchor and the evaluated structures. As a consequence, deviations are expected to increase with growing distance from the reference structure due to the accumulation of matching errors along the scan trajectory [4, 8, 27].

The present results confirm this concept, as identical scan datasets yielded different trueness and precision values solely depending on the spatial location of the reference body used for alignment. This indicates that the matching and stitching process is locally optimized around the chosen reference structure, while systematic distortions become more pronounced with increasing distance from this anchor point.

The observed differences can plausibly be explained by the propagation of matching and stitching errors along the scan path in combination with the anchoring effect of the selected reference coordinate system. Importantly, these effects were consistently present for both investigated intraoral scanners, suggesting that the influence of reference body position represents a general methodological phenomenon rather than a scanner-specific characteristic.

### Implications for the comparability of accuracy studies

Reference structures such as cuboids or geometric markers are frequently used in in vitro accuracy studies to establish a coordinate system and to enable absolute measurements [9, 17, 28, 29]. However, their position within the model is usually selected pragmatically and rarely considered as an independent experimental variable. The present findings demonstrate that the reference body location itself can introduce systematic deviations and therefore represents a potential source of bias when comparing accuracy data across different studies.

Consequently, studies employing reference structures at different positions within the model may not be directly comparable, even if identical scanners, scan bodies, and evaluation methods are used. This aspect is particularly relevant for systematic reviews and meta-analyses, in which heterogeneity of reported accuracy values is frequently observed and often attributed solely to differences in scanner technology or scanning strategy [2, 30]. The present results suggest that variations in reference system positioning may additionally contribute to this variability.

The present findings therefore raise a fundamental methodological question regarding the comparability of accuracy studies. Even when identical scan datasets and identical measurement points are evaluated, the use of different reference body positions can lead to significantly different trueness and precision values. This implies that differences reported between studies may not only be attributed to scanner technology, scanning strategy, or evaluation software, but also to the spatial location of the reference system used to establish the coordinate framework [6, 23, 28, 29, 31].

### Influence of reference body geometry

The reference bodies used in the present study consisted of geometric cuboids that do not exhibit dental morphology. It might therefore be assumed that feature detection and matching could differ from those of natural tooth surfaces or scan bodies [24, 28]. Nevertheless, systematic differences between REF-1 and REF-2 were consistently observed for both scanners and for both linear and 3D measurements. This indicates that the spatial position of the reference structure and the resulting coordinate anchoring appear to have a stronger influence on the alignment outcome than the specific surface morphology of the reference body itself.

### Clinical relevance

It should be emphasized that the observed reference body-dependent effects are primarily relevant for the assessment of accuracy and the comparability of in vitro studies. In clinical intraoral scanning procedures, where such artificial reference bodies are typically not present, this specific source of bias does not directly occur.

Implants exhibit substantially lower intrinsic mobility than natural teeth and therefore do not allow for physiological compensation of spatial inaccuracies [18, 32]. As a result, even small systematic deviations in implant position may be clinically relevant for the fit of implant-supported prostheses. The present findings indicate that reference-dependent alignment effects can influence both trueness and precision of measured implant positions, which should be considered when interpreting the accuracy of digital impressions and when comparing different studies or digital workflows.

### Limitations

This study was performed under in vitro conditions using a single model and two IOS systems. Only two reference body positions were evaluated, and a single scan path strategy was applied. Different scanning strategies were not investigated in the present study. As scan path length and direction are known to influence stitching errors and cumulative deviations, future studies should investigate whether the observed reference-body-dependent effects are affected by variations in scanning strategy. Furthermore, the reference structures consisted of standardized cuboids and did not represent anatomical tooth morphology. Although standardized cuboid-shaped reference bodies were used in the present study, similar reference-dependent effects may also be expected when using anatomical structures or scan bodies, as the underlying mechanism is primarily related to spatial anchoring and error propagation rather than surface morphology.

Therefore, the results cannot be directly transferred to all clinical situations. Nevertheless, the controlled in vitro setup allowed isolation of the reference body position as an independent variable and enabled a systematic investigation of its influence on alignment and measurement accuracy.

## Conclusions

The present study demonstrates that the position of the reference body within a model significantly influences the accuracy (trueness and precision) of both linear and 3D measurements of implant positions, even when identical intraoral scan datasets are analyzed. These findings highlight the importance of considering reference system positioning when designing, interpreting, and comparing accuracy studies in digital implant dentistry, as reference body location represents an independent source of systematic bias.

## Data Availability

Data is provided by corresponding author on reasonable request.

## Abbreviations

3D: Three-dimensional
FDI: Federation dentaire internationale
IAIPs: Implant abutment interface points
IMM: Implant master model
ISO: International Organization for Standardization
STL: Standard tessellation language
